# Tolerant and Rapid Endochondral Bone Regeneration Using Framework‐Enhanced 3D Biomineralized Matrix Hydrogels

**DOI:** 10.1002/advs.202305580

**Published:** 2023-12-21

**Authors:** Baoshuai Bai, Yanhan Liu, Jinyi Huang, Sinan Wang, Hongying Chen, Yingying Huo, Hengxing Zhou, Yu Liu, Shiqing Feng, Guangdong Zhou, Yujie Hua

**Affiliations:** ^1^ Shanghai Key Laboratory of Tissue Engineering Department of Plastic and Reconstructive Surgery of Shanghai Ninth People's Hospital Shanghai Jiao Tong University School of Medicine Shanghai 200011 P. R. China; ^2^ National Tissue Engineering Center of China Shanghai 200241 P. R. China; ^3^ Department of Orthopaedics Advanced Medical Research Institute Qilu Hospital of Shangdong University Centre for Orthopaedics Shandong University Jinan Shandong 250100 P. R. China; ^4^ Department of Orthopaedics Cheeloo College of Medicine The Second Hospital of Shandong University Shandong University Jinan Shandong 250033 P. R. China; ^5^ Department of Ophthalmology Renji Hospital School of Medicine Shanghai Jiao Tong University Shanghai 200127 P. R. China

**Keywords:** biomineralization, bone regeneration, endochondral ossification, hydrogels, hypoxic microenvironment

## Abstract

Tissue‐engineered bone has emerged as a promising alternative for bone defect repair due to the advantages of regenerative bone healing and physiological functional reconstruction. However, there is very limited breakthrough in achieving favorable bone regeneration due to the harsh osteogenic microenvironment after bone injury, especially the avascular and hypoxic conditions. Inspired by the bone developmental mode of endochondral ossification, a novel strategy is proposed for tolerant and rapid endochondral bone regeneration using framework‐enhanced 3D biomineralized matrix hydrogels. First, it is meticulously designed 3D biomimetic hydrogels with both hypoxic and osteoinductive microenvironment, and then integrated 3D‐printed polycaprolactone framework to improve their mechanical strength and structural fidelity. The inherent hypoxic 3D matrix microenvironment effectively activates bone marrow mesenchymal stem cells self‐regulation for early‐stage chondrogenesis via TGFβ/Smad signaling pathway due to the obstacle of aerobic respiration. Meanwhile, the strong biomineralized microenvironment, created by a hybrid formulation of native‐constitute osteogenic inorganic salts, can synergistically regulate both bone mineralization and osteoclastic differentiation, and thus accelerate the late‐stage bone maturation. Furthermore, both in vivo ectopic osteogenesis and in situ skull defect repair successfully verified the high efficiency and mechanical maintenance of endochondral bone regeneration mode, which offers a promising treatment for craniofacial bone defect repair.

## Graphical abstract

In this study, a tolerant and rapid endochondral bone regeneration strategy is proposed using 3D‐printed PCL framework‐enhanced biomineralized matrix hydrogels with both hypoxic and osteoinductive microenvironment, which can effectively regulate BMSC‐based endochondral ossification (**ECO**) for tissue‐engineered bone construction. The current study eventually realized satisfactory ectopic osteogenesis in nude mice and skull defect repair in rabbits by the high‐efficiency **ECO** mode.

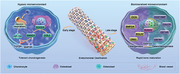

## 1 Introduction

Bone repair materials, such as metal, ceramic, and polymer‐based artificial materials, play an essential role in treating bone defects.^[^ ^1^, ^2^, ^3^, ^4^, ^5^, ^6^ ^]^ Although these clinically therapeutic treatments could satisfactorily realize the basic requirements of defect filling and mechanical support, they commonly limit to non‐regenerative bone healing without physiologically functional recovery.^[^ ^7^, ^8^, ^9^, ^10^, ^11^ ^]^ Tissue‐engineered (**TE**) bone, combining seed cells with biocompatible scaffolds, has emerged as an alternative treatment due to the advantages of regenerative bone healing and functional reconstruction.^[^ ^12^, ^13^, ^14^, ^15^, ^16^, ^17^, ^18^, ^19^ ^]^ Recently, our groups have pioneered to propose a framework‐enhanced **TE** bone construction strategy by means of 3D‐printed polycaprolactone (**PCL**) or decalcified bone scaffolds to overcome the inherent drawbacks of inferior mechanical properties and morphological fidelity, which could successfully realize craniofacial or long bone defect repair in physiological microenvironment.^[^ ^20^, ^21^ ^]^ However, there has seen very limited breakthrough in achieving favorable bone regeneration when occurred in pathological microenvironment, especially the avascular and hypoxic conditions.

Generally, bone regeneration modes present two distinct approaches: intramembranous ossification (**IMO**) and endochondral ossification (**ECO**).^[^ ^22^, ^23^, ^24^, ^25^ ^]^ For most craniofacial and long bones, previous researches have reported that the **IMO** mode often leads to avascular necrosis and degradation in central regions due to insufficient angiogenesis and poor nutrient perfusion, particularly in harsh microenvironment.^[^ ^24^, ^26^, ^27^, ^28^ ^]^ In comparison, the **ECO** mode, a time‐dependent process starting with an initially cartilaginous template followed by hypertrophy and mineralization, is extremely tolerant to avascular and hypoxic microenvironments due to the early‐stage chondrogenic formation.^[^ ^29^, ^30^, ^31^ ^]^ Inspired by the bone developmental mode of **ECO**, we speculated that adopting the **ECO** mode would effectively enable it to withstand the harsh microenvironment. To realize this strategy, it is essential to develop suitable scaffolds for precisely regulating endochondral bone regeneration.

As known, traditional 2D culture relies on cells seeding onto the polymer‐based scaffolds,^[^ ^32^, ^33^ ^]^ whereas the current 3D culture depends on cells encapsulating into the hydrogel‐based scaffolds.^[^ ^34^, ^35^, ^36^ ^]^ Unlike the oxygen‐rich and well‐nutritional system of 2D culture, cells in the high‐density 3D hydrogel‐based matrix are highly restricted that is considered as a hypoxic microenvironment hindering oxygen exchange and vascular infiltration.^[^ ^37^, ^38^ ^]^ Noticeably, the previous studies demonstrated that the hypoxic microenvironment could induce the chondrogenic differentiation of bone marrow mesenchymal stem cells (BMSCs) by activating the hypoxia‐inducible factor 1‐α (HIF‐1α) signaling pathway.^[^ ^39^, ^40^, ^41^ ^]^ Therefore, it is reasonable to speculate that BMSCs in 3D hydrogels would undergo spontaneous chondrogenic development regulated by the hypoxic microenvironment, but the hydrogel‐based molecular mechanism has not been clearly revealed. To construct **TE** bone via the **ECO** mode, another consideration is to synergistically provide an ideal osteoinductive microenvironment. Despite the prevalent use of bioactive growth factors, osteogenic inorganic salts, such as hydroxyapatite, calcium silicate, calcium citrate, magnesium phosphate, and zinc phosphate, have the special advantages of low costs, long‐term storage capabilities, and stable bioactive maintenance.^[^ ^14^, ^20^, ^38^, ^42^ ^]^ Furthermore, native‐constituent osteogenic inorganic salts (**NOIS**) consist of a hybrid formulation of multiple biomineralized elements, which could strike a balance between the bone regeneration and remodeling process involving the deposition of new bone and reabsorption of old bone. Taken together, it is necessary to combine the hypoxic and osteoinductive microenvironments simultaneously to achieve BMSC‐based endochondral bone regeneration.

**Figure 1:**
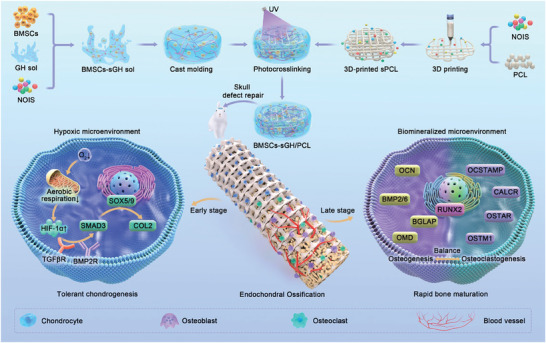
Schematic illustration of a tolerant and rapid endochondral bone regeneration strategy using framework‐enhanced 3D biomineralized matrix hydrogels. BMSCs, bone marrow stem cells; **NOIS**, native‐constituent osteogenic inorganic salt; **GH**, GelMA/HAMA; **sGH**, NOIS@GelMA/HAMA; **PCL**, polycaprolactone; **sPCL**, NOIS@PCL.

Herein, we developed a promising bone regeneration mode of spontaneous **ECO** using 3D biomineralized matrix hydrogels with both innate hypoxic microenvironment and strong osteoinductive microenvironment (**Figure** **1**). In the current design, unlike 2D cell culture, 3D matrix hydrogels that contained the native tissue‐like constitution of both proteins and glycosaminoglycans possess hypoxic microenvironment to hinder aerobic respiration, and thus facilitate the positive regulation of cartilage development by activating TGFβ/Smad signaling pathway. Meanwhile, the osteogenic inorganic salts could offer biomineralized microenvironment to make a balance between regulation of bone mineralization and osteoclastic differentiation, which would accelerate the mature rate of bone regeneration. To further improve the mechanical strength and structural fidelity, a 3D‐printed **PCL** framework was employed to integrate with biomineralized matrix hydrogels. Finally, the feasibility of BMSC‐based bone regeneration via the **ECO** mode was verified using both an ectopic osteogenesis model in nude mice and an in situ osteogenesis model for repairing skull defects in rabbits. Overall, this study thus illustrates a tolerant and rapid endochondral bone regeneration mode, which pioneered a promising approach for craniofacial bone defect repair.

## 2 Results

### 2.1 Characterization of Framework‐Enhanced Biomineralized Matrix Hydrogels

**Figure 2:**
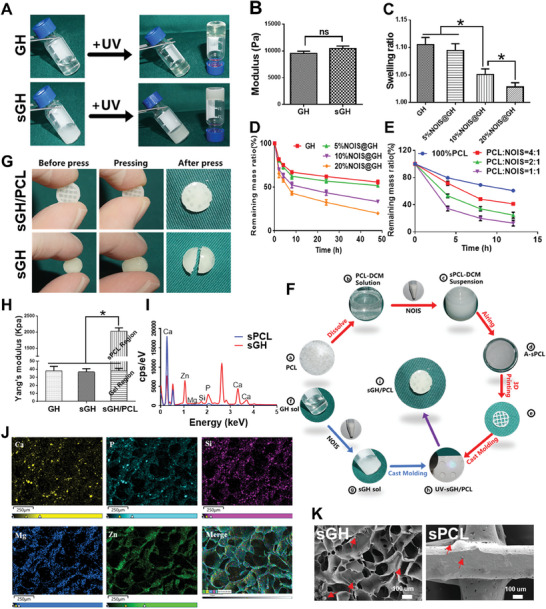
Characterization of framework‐enhanced biomineralized matrix hydrogels. A,B) Photographs (A) and rheological data (B) of **GH** and **sGH** hydrogels before and after 365 nm light irradiation. Data are presented as mean ± standard deviations (SD), n = 3, ns: not statistically significant. C–E) The swelling ratio (C) and degradation rate of **sGH** hydrogels (D) and the **sPCL** framework (E) with different **NOIS** contents. Data are presented as mean ± SD, n = 3, ^*^: *p* < 0.05. F) The preparation process of 3D‐printed **sPCL**‐enhanced sGH hydrogel scaffolds. G) A comparison of **sGH** and **sGH/PCL** scaffolds before and after pressing. H) The compressive modulus of **GH**, **sGH**, and **sGH/PCL** scaffolds. Data are presented as mean ± SD, n = 3, ^*^: *p* < 0.05. I,J) Elemental analyses (I) and photographs (J) of the **sGH** hydrogels and **sPCL** framework. K) Scanning electron microscopy (SEM) images of the **sGH** hydrogels and **sPCL** framework. Red arrows indicate the existence of inorganic salts. **NOIS**, native‐constituent osteogenic inorganic salt; **GH**, 5% (w/v) GelMA/1% (w/v) HAMA; **sGH**, 5% (w/v) NOIS@ 5% (w/v) GelMA/1% (w/v) HAMA; **sPCL**, 20% (w/v) NOIS@PCL (PCL: NOIS = 4:1); light, 365 nm LED, 20 mW cm^−2^.

To create biomineralized matrix hydrogels, protein‐contained gelatin methacryloyl (GelMA) and glycosaminoglycan‐contained hyaluronic acid methacryloyl (HAMA) are chosen as matrix biomimetic components, which is conducive to mimic the native tissue‐like constitution consisted of both proteins and glycosaminoglycans. Meanwhile, **NOIS**, that is, hydroxyapatite, calcium silicate, magnesium phosphate, and zinc phosphate, are chosen as biomineralized components that characterized by SEM examination respectively (Figure S1, Supporting Information). First, the sol‐to‐gel transition of both GelMA/HAMA (**GH)** and NOIS@GelMA/HAMA (**sGH)** hydrogels occurred rapidly upon light irradiation (365 nm LED, 20 mW cm^−2^) through a photoinitiated polymerization reaction, of which the physicochemical structure was characterized by Fourier transform infrared (FTIR) spectroscopy (**Figure** **2**A,B; Figure s2 , Supporting Information). Noticeably, the addition of native‐constitute osteogenic inorganic salts into the **GH** hydrogels has no significant influence on the neutral pH value of gel precursor solution (**GH** sol: pH 7.34 ± 0.08; **sGH** sol: pH 7.46 ± 0.14). To determine a reliable ratio of **NOIS** in both **GH** hydrogels and the **PCL** framework, the swelling ratio and degradation rate were assessed. As shown in Figure 2C–E, the inclusion of 5% (w/v) **NOIS** in the biomineralized matrix hydrogels and 20% (w/v) **NOIS** (**PCL**: **NOIS** = 4:1) in the **PCL** framework did not significantly influence these physiochemical characteristics when compared to non‐**NOIS** scaffolds. These findings indicate that the **sGH** hydrogels exhibited a relatively low swelling ratio (110.5% ± 0.74%) and could be gradually degraded under enzymolysis conditions.

To further enhance the mechanical strength of **sGH** hydrogels, a 3D‐printed **sPCL** was integrated with the hydrogels based on the “steel **sPCL**‐reinforced concrete **sGH**” design principle. The preparation process of the **sGH/PCL** scaffolds is illustrated in Figure 2F: initially, **PCL** a) was dissolved in dichloromethane (DCM) to form a **PCL**‐DCM solution b). **NOIS** was then added to the **PCL**‐DCM solution to form a **sPCL**‐DCM suspension c), which was subsequently air‐dried to obtain A‐**sPCL** d). Extrusion‐based 3D printing was used to prepare the latticed 3D‐printed **sPCL** e). Meanwhile, **NOIS** was added to **GH** sol f) to obtain **sGH** sol g). The **sGH/PCL** scaffolds i) were then prepared and integrated using a photo‐crosslinking approach h). As shown in Figure 2G, the introduce of **sPCL** significantly enhanced the mechanical strength of the **sGH** hydrogels, enabling them to effectively withstand applied forces. Quantitative analyses of the Yang's modulus demonstrated that the mechanical strength of the **sGH/PCL** scaffolds (1987.0 ± 61.1 kPa) was ≈55 times higher than that of the **sGH** hydrogels (36.5 ± 2.4 kPa), with the **NOIS** content (5% w/v **NOIS** for **sGH** hydrogels; 20% w/v **NOIS** for **sPCL** scaffolds) optimized to achieve the best formulation (Figure 2H; Figure S3, Supporting Information). Furthermore, the presence of metal elements such as calcium, phosphorus, magnesium, zinc, and silicon were identified in both the **sGH** hydrogels and the **sPCL** framework (Figure 2I,J,; Figure S4, Supporting Information), confirming the native constituents of osteogenic inorganic salts. In addition, scanning electron microscopy (SEM) images showed the surface morphology of the **sGH** and **sPCL**, further supporting the existence of **NOIS** in both the **sGH** hydrogels and the **sPCL** framework (Figure 2K).

### 2.2 In Vitro Cytocompatibility and Biological Function Evaluation

**Figure 3:**
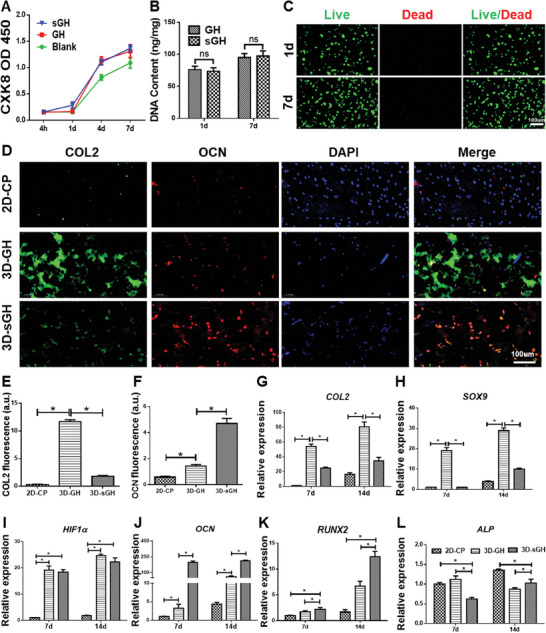
In vitro cytocompatibility and biological function evaluation. A) Cytotoxicity analyses of **GH** and **sGH** hydrogels at 4 h, as well as 1, 4, and 7 days. B,C) DNA content and Live/dead staining of BMSCs in the **sGH** hydrogels after 1‐ and 7‐days culture in vitro. Data are presented as mean ± SD, n = 3, ns: not statistically significant. D) Immunofluorescence staining of COL2 and OCN in **2D‐CP**, **3D‐GH**, and **3D‐sGH** groups at 14 days. E,F) Quantitative analyses of COL2 (E) and OCN (F) fluorescence intensity. Data are presented as mean ± SD, n = 3, ^*^: *p* < 0.05. G–K) Expression level of cartilage‐related genes (*COL2*, *SOX9*), hypoxia‐inducible gene (*HIF‐1α*), and bone‐related genes (*OCN*, *RUNX2*) in **2D‐CP**, **3D‐GH**, and **3D‐sGH** groups after 7‐ and 14‐days culture in vitro. Data are presented as mean ± SD, n = 3, ^*^: *p* < 0.05. The scaffold compositions are the same as shown in Figure 2.

To verify the cytocompatibility, CCK‐8 and DNA content assays, as well as live/dead staining, were performed in vitro on both the 2D culture platform (**2D‐CP**) and the 3D hydrogel culture platform (**3D‐GH** and **3D‐sGH** groups). The cytotoxicity analysis revealed that the addition of **NOIS** to the **GH** gel precursor did not significantly affect cell proliferation, implying that both **NOIS** and the **GH** gel precursor were basically nontoxic (**Figure** **3**A,B). Furthermore, cell viability and cytoskeleton assays demonstrated that BMSCs grew well in the **3D‐GH** and **3D‐sGH** hydrogels with minimal cell death and limited spreading behavior after one and seven days of culture in vitro, indicating favorable cytocompatibility of the **GH** and **sGH** hydrogels (Figure 3C; Figures S5 and s6 , Supporting Information). To investigate the biological functions of the hydrogel scaffolds and biomineralized salts, immunofluorescence staining and real‐time polymerase chain reaction (RT‐PCR) analyses were performed. Immunofluorescence staining and corresponding quantitative analyses between the **2D‐CP** and **3D**‐**GH** groups revealed that the expression of the cartilage‐specific protein COL2 was obviously upregulated in the **3D‐GH** group compared to the **2D‐CP** group (Figure 3D,E), which was further confirmed by the expression of chondrogenic genes *COL2* and *SOX9*, as well as the hypoxia‐inducible gene *HIF‐1α* (Figure 3G–I). Additionally, the expression of the bone‐specific protein OCN in the **3D‐GH** and **3D‐sGH** groups indicated that the addition of **NOIS** effectively promoted BMSC osteogenesis (Figure 3D,F). This was further confirmed by the expression of osteogenic genes *OCN, RUNX2*, and *ALP* (Figure 3J–L).

### 2.3 Molecular Mechanism of Endochondral Ossification Mode

**Figure 4:**
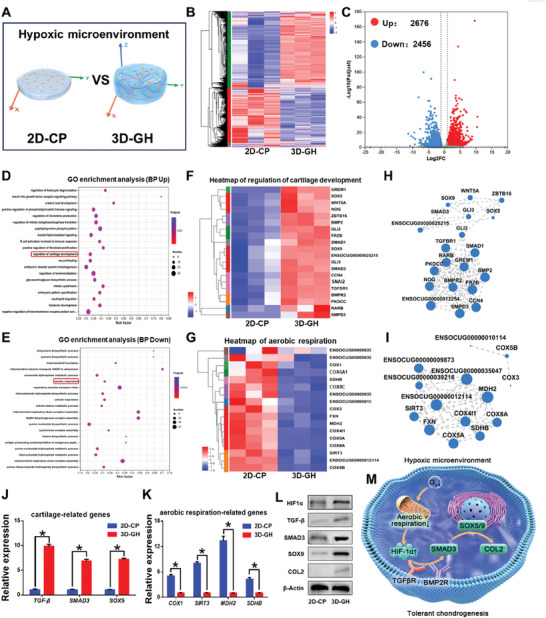
Molecular mechanisms underlying spontaneous chondrogenesis in 3D matrix hydrogels. A) Schematic illustration of stem cell fate in 2D and 3D culture platforms. B,C) Heatmap and volcano plot of differentially expressed genes (DEGs) between the **2D‐CP** and **3D‐GH** groups. D,E) Gene ontology (GO) enrichment analyses of upregulated (D) and downregulated (E) DEGs for total biological processes. F,G) Heatmaps of biological processes involving “regulation of cartilage development” (F) and “aerobic respiration” (G) (marked in red) in the corresponding GO enrichment. H,I) Gene expression correlation analyses of biological processes involving “regulation of cartilage development” (H) and “aerobic respiration” (I). J,K) Real‐time polymerase chain reaction (RT‐PCR) results of the expression levels of cartilage‐related genes *TGF‐β*, *SMAD3*, and *SOX5*, as well as aerobic respiration‐related genes *COX1*, *SIRT3*, *MDH*, and *SDHB*. Data are presented as mean ± SD, n = 3, ^*^: *p* < 0.05. L) Weston blot analysis of the expression levels of cartilage‐related proteins HIF‐1α, SOX9, COL2, TGF‐β, and SMAD3. M) Schematic illustration of spontaneous chondrogenesis via the transforming growth factor beta (TGF‐β)/Smad signaling pathway.

To elucidate the molecular mechanism underlying spontaneous chondrogenesis in 3D matrix hydrogels, transcriptome sequencing of BMSCs after 14 days of culture was conducted to systematically compare gene expressions between the **2D‐CP** and **3D‐GH** groups (**Figure** **4**A). The heatmap and volcano plot revealed 2476 upregulated and 2656 downregulated differentially expressed genes (DEGs) between the 2D and 3D culture platforms, implying the unique 3D matrix microenvironment provided by the **3D‐GH** groups (Figure 4B,C). The gene ontology (GO) enrichment analysis of the upregulated DEGs demonstrated the essential biological process of “regulation of cartilage development” (marked in red), indicating the accessibility to spontaneous chondrogenesis in 3D matrix hydrogels (Figure 4D). Subsequently, clustering and gene expression correlation analyses were performed to evaluate the biological process of “regulation of cartilage development” and identify the related DEGs and their internal relationship to cartilage development (Figure 4F,H). Similarly, the GO enrichment analysis of the downregulated DEGs, that is, low gene expression in the **3D‐GH** groups, revealed the key biological process of “aerobic respiration” (marked in red). This is consistent with the speculation of insufficient oxygen supply in 3D matrix hydrogels compared to the 2D culture platform (Figure 4E). Clustering and gene expression correlation analyses further confirmed significant targeted genes and their internal relationships involved in “aerobic respiration” (Figure 4G,I). Furthermore, RT‐PCR was performed to verify the upregulation of the expression levels of cartilage‐related genes *TGF‐β*, *SMAD3*, and *SOX5* (Figure 4J). Meanwhile, the expression levels of cartilage‐related proteins HIF‐1α, SOX9, COL2, TGF‐β, and SMAD3 were also confirmed by western blot analysis, indicating that BMSCs in 3D matrix hydrogels were prone to chondrogenesis via the TGF‐β/Smad signaling pathway (Figure 4L). In terms of the aerobic respiration process, the expression levels of the related genes *COX1*, *SIRT3*, *MDH*, and *SDHB* were significantly downregulated, confirming the results of the GO enrichment analysis (Figure 4K). Taken together, the inherent hypoxic 3D matrix microenvironment can effectively activate HIF‐1α self‐regulation by limiting aerobic respiration, thereby facilitating spontaneous chondrogenesis via the TGF‐β/Smad signaling pathway (Figure 4M).

**Figure 5:**
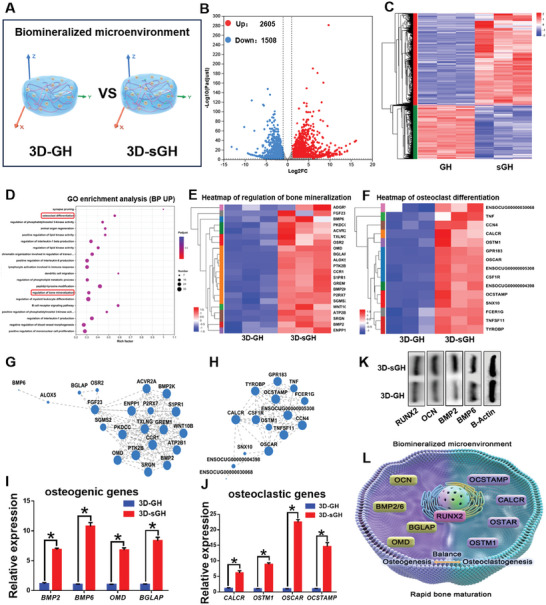
Molecular mechanisms underlying efficient osteogenesis in 3D biomineralized matrix hydrogels. A) Schematic illustration of stem cell fate with and without the addition of biomineralized inorganic salts in the 3D culture platform. B,C) Heatmap and volcano plot of DEGs between the GH and Sgh groups. D) GO enrichment analyses of upregulated DEGs for total biological processes. E,F) Heatmaps of biological processes involving “regulation of bone mineralization” and “osteoclast differentiation” (marked in red) in the corresponding GO enrichment. G,H) Gene expression correlation analyses of biological processes involving “regulation of bone mineralization“ (G) and ”osteoclast differentiation" (H). I,J) RT‐PCR results of the expression levels of osteogenic genes *BMP2*/*6*, *OMD*, and *BGLAP* (I), as well as osteoclastic genes *CALCR*, *OSTM1*, *OSCAR*, and *OCSTAMP* (J). Data are presented as mean ± SD, n = 3, ^*^: *p* < 0.05. K) Weston blot analysis of the expression levels of bone‐related proteins RNX2, OCN, and BMP2/6. L) Schematic illustration of efficient osteogenesis achieved via striking a balance between bone formation and remodeling processes.

To further elucidate the efficient osteoinductive molecular mechanism of biomineralized inorganic salts, transcriptome sequencing of BMSCs after 14‐days culture was performed to systematically compare gene expressions between the **3D‐GH** and **3D‐sGH** groups (**Figure** **5**A). The heatmap and volcano plot revealed 2605 upregulated and 1508 downregulated DEGs between the **3D‐GH** and **3D‐sGH** groups, explaining the obvious effect upon **NOIS** addition (Figure 5B,C). The GO enrichment analysis of the upregulated DEGs demonstrated bone‐related biological processes such as “regulation of bone mineralization” and “osteoclast differentiation” (marked in red), suggesting that **NOIS** addition in **GH** hydrogels could effectively promote bone maturation (Figure 5D). Subsequently, clustering and gene expression correlation analyses were performed to evaluate the biological processes of “regulation of bone mineralization” and “osteoclast differentiation” and identify significant targeted genes and their internal relationship to bone development (Figure 5E–H). Furthermore, RT‐PCR was performed to verify the upregulation of the expression levels of osteogenic genes *BMP2*/*6*, *OMD*, and *BGLAP*, as well as osteoclastic genes *CALCR*, *OSTM1*, *OSCAR*, and *OCSTAMP*. These findings demonstrated efficient bone maturation via striking a balance between bone formation and remodeling processes (Figure 5I,J). Meanwhile, the expression levels of bone‐related proteins RUX2, OCN, and BMP2/6 were further confirmed using Weston blot analysis, indicating that BMSCs in 3D biomineralized matrix hydrogels have the ability to secrete osteogenic proteins for bone formation (Figure 5K). Therefore, the strong biomineralized microenvironment could synergistically regulate both bone mineralization and osteoclastic differentiation and thus effectively accelerate bone maturation (Figure 5L).

### 2.4 In Vivo Endochondral Bone Regeneration in Nude Mice

**Figure 6:**
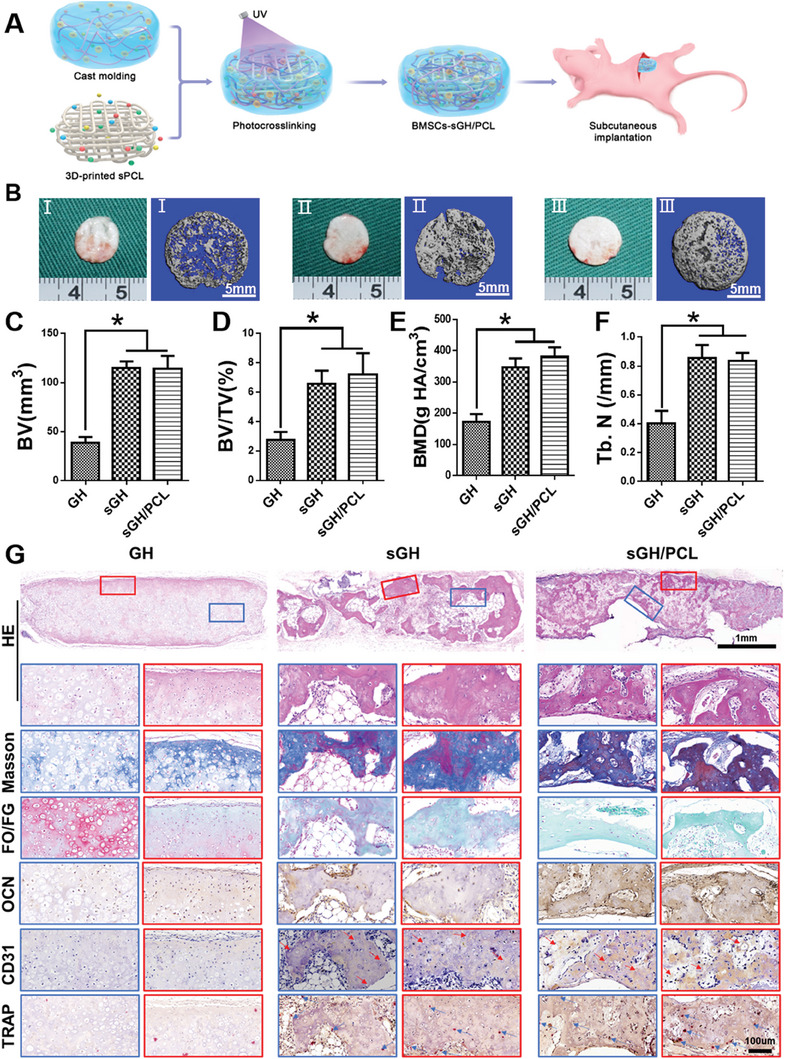
Evaluation of regenerated bone at four weeks post‐implantation in nude mice. A) Schematic illustration of the 3D‐printed framework‐enhanced biomineralized matrix hydrogels. B) The gross views and micro‐CT images of the **GH**, **sGH**, and **sGH/PCL** groups. C–F) Quantitative analyses of bone volume (BV) (C), bone volume fraction (BV/TV) (D), bone mineral density (BMD) (E), and the number of bone trabeculae (Tb.N) (F). Data are presented as mean ± SD, n = 3, ^*^: *p* < 0.05. G) Histological examinations of hematoxylin and eosin (H&E), safranin‐O/fast green (SO/FG), Masson, Type II collagen (COL2), osteocalcin (OCN), CD31, and tartrate‐resistant acid phophatase (TRAP) staining in the **GH**, **sGH**, and **sGH/PCL** groups. The red arrows represent new vessels. The scaffold compositions are the same as those shown in Figure 2.

**Figure 7:**
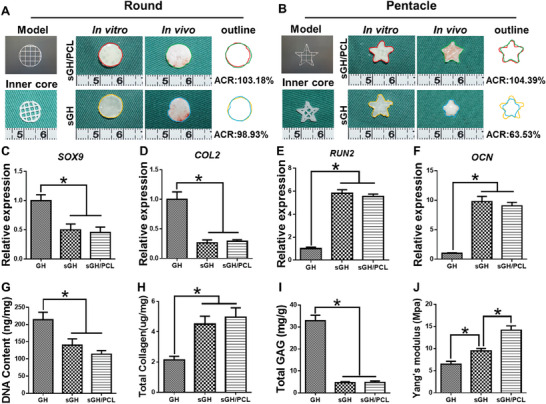
Shape maintenance and quantitative analyses of regenerated bone in nude mice. A,B) Round and pentacle shape maintenance of the **sGH** and **sGH/PCL** groups four weeks after implantation in nude mice. C–F) Expression levels of cartilage‐related genes *COL2* and *SOX9*, as well as bone‐related genes *OCN* and *RUNX2*, in the **GH**, **sGH**, and **sGH/PCL** groups four weeks after implantation. Data are presented as mean ± SD, n = 3, ^*^: *p* < 0.05. G–J) Quantitative analyses of the DNA content (G), GAG content (H), total collagen (I), and Yang's modulus (J) in the **GH**, **sGH**, and **sGH/PCL** groups four weeks after implantation. Data are presented as mean ± SD, n = 3, ^*^: *p* < 0.05. The scaffold compositions are the same as those shown in Figure 2. ACR, area change rate.

To further evaluate in vivo endochondral bone regeneration, BMSCs‐loaded **GH**, **sGH**, and **sGH/PCL** were subcutaneously implanted in nude mice. Based on the design concept of the “steel‐reinforced concrete” principle, **sPCL** (steel) was used to reinforce the mechanical strength and structural fidelity of sGH hydrogels (concrete) for bone regeneration (**Figure** **6**A). The samples were harvested four and eight weeks after implantation. Gross views and micro‐CT examinations revealed that the regenerated bone tissue in both the **sGH** and **sGH/PCL** groups displayed a larger mineralized area than that of the **GH** groups, implying that the addition of **NOIS** could effectively promote mineralization for bone regeneration (Figure 6B). In addition, the integration of 3D‐printed **sPCL** had no significant influence on the degree of mineralization, as indicated by the similar mineralized area between the **sGH** and **sGH/PCL** groups. This finding was further confirmed by quantitative analyses of bone volume (BV), bone volume fraction (BV/TV), bone mineral density (BMD), and number of bone trabeculae (Tb.N) for bone regeneration (Figure 6C–F). Furthermore, the round shape of regenerated bone tissue (simple shape) was well‐maintained with or without **sPCL** inner support (**sGH** group: ≈98.9%; **sGH/PCL** group: ≈103.2%). However, the pentacle shape of regenerated bone tissue (complex shape) required the assistance of **sPCL** inner support to maintain structural fidelity (**sGH** group: ≈104.4%; **sGH/PCL** group: ≈63.5%). These findings indicate that **sPCL** inner support for **sGH** hydrogel scaffolds is necessary for repairing irregular, larger‐sized bone defects (**Figure** **7**A,B; Figure S7, Supporting Information).

Furthermore, histological examinations revealed the presence of cartilaginoid tissue with cartilage‐specific staining for safranin‐O in the **GH** groups, which was in sharp contrast to the **sGH** and **sGH/PCL** groups. As shown in Figure 6G, both the **sGH** and **sGH/PCL** groups showed bone‐like tissue with a similar mineralized area and vascularized bone‐specific protein deposition, as demonstrated by Masson's trichrome, fast green, osteocalcin (OCN), and CD31 staining, as well as osteoclast differentiation, as demonstrated by tartrate‐resistant acid phosphatase (TRAP) staining. Eight weeks after implantation, histological examinations showed a similar extent of osteogenesis in the **GH**, **sGH**, and **sGH/PCL** groups (Figure S8, Supporting Information), indicating that the addition of **NOIS** could accelerate the process of bone maturation at four weeks. Quantitative analyses further validated the differences in bone regeneration among the **GH**, **sGH**, and **sGH/PCL** groups. The expression levels of cartilage‐specific genes *SOX9* and *COL2* in the **GH** group were significantly upregulated compared to the **sGH** and **sGH/PCL** groups (Figure 7C,D). Additionally, the **GH** group exhibited a higher content of DNA and GAG, as well as a lower content of total collagen (Figure 7G–I). Meanwhile, the expression levels of bone‐specific genes *RUX2* and *OCN* in both the **sGH** and **sGH/PCL** groups were significantly upregulated compared to the GH group (Figure 7E,F). These findings, along with the higher content of total collagen and lower content of DNA and GAG, suggest that the addition of **NOIS** into GH hydrogel scaffolds can effectively promote osteogenesis (Figure 7G–I). Furthermore, there were no significant differences between the **sGH** and **sGH/PCL** groups in terms of gene expression level, DNA content, total collagen, and GAG. However, the Yang's modulus of the **sGH/PCL** groups was obviously higher than that of the **sGH** groups (Figure 7J), indicating the addition of **sPCL** to **sGH** hydrogel scaffolds could reinforce the mechanical strength and structural stability of the regenerated bone.

### 2.5 In Situ Repair of Rabbit Skull Defects

**Figure 8:**
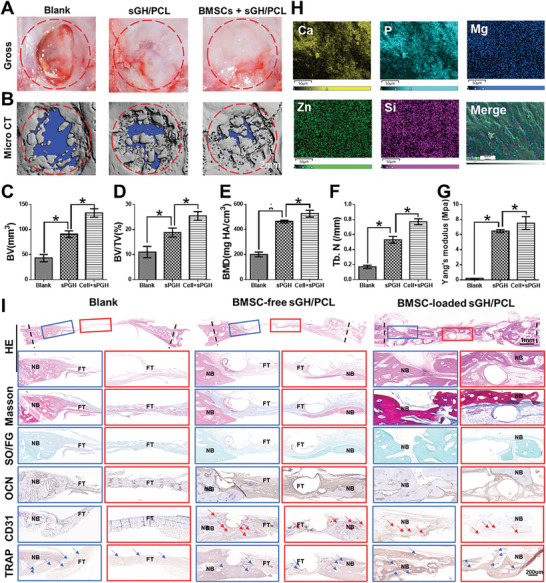
Evaluation of rabbit skull defect repair at 12 weeks post‐surgery. A,B) Gross view (A) and 3D reconstruction microcomputed tomography scan images (B) of the bone marrow mesenchymal stem cells (BMSCs)‐loaded **sGH/PCL**, BMSCs‐free **sGH/PCL**, and blank groups. C–G) Quantitative analyses of bone volume (BV) (C), bone volume fraction (BV/TV) (D), bone mineral density (BMD) (E), number of bone trabeculae (Tb.N) (F), and the Yang's modulus (G) in the BMSCs‐loaded **sGH/PCL**, BMSCs‐free **sGH/PCL**, and blank groups. Data are presented as mean ± SD, n = 3, ^*^: *p* < 0.05. H) Elemental analyses of regenerated skull defects in the BMSCs‐loaded **sGH/PCL** groups. I) Histological examinations of H&E, SO/FG, Masson, OCN, CD31, and TRAP staining in the BMSCs‐loaded **sGH/PCL**, BMSCs‐free **sGH/PCL**, and blank groups. The scaffold compositions are the same as those shown in Figure 2.

Finally, a critical aspect to further clarify the clinical and translational potential of the current technique is to assess whether the framework‐enhanced 3D biomineralized matrix hydrogel scaffolds can effectively repair craniofacial bone defects. To investigate the role of **sGH/PCL** scaffolds and exogenous BMSCs in repairing rabbit skull defects, BMSCs‐loaded and BMSC‐free **sGH/PCL** groups were compared, with untreated skull defects serving as the blank group. As shown in **Figure** **8**A–F, the gross view, micro‐CT scan images, and corresponding quantitative analyses (BV, BV/TV, BMD, and Tb.N) demonstrated that the BMSCs‐loaded **sGH/PCL** groups exhibited the largest mineralized area in the regenerated skull, followed by the BMSCs‐free **sGH/PCL** and blank groups. The regeneration effect of skull defects was further supported by quantitative data on Yang's modulus and elemental analyses of calcium, phosphorus, magnesium, zinc, and silicon, indicating satisfactory bone regeneration and mineralization in the BMSCs‐loaded **sGH/PCL** groups (Figure 8G,H; Figure S9, Supporting Information). Furthermore, histological examinations revealed that the blank group achieved partial bone regeneration with predominantly fibrous tissue ingrowth. The BMSCs‐free **sGH/PCL** group exhibited more mineralization and less fibrous tissue. However, the BMSCs‐loaded **sGH/PCL** group demonstrated the most effective repair with vascularized bone regeneration (OCN and CD31 staining) and osteoclastic differentiation (TRAP staining), thereby accelerating the process of bone maturation (Figure 8I). These findings demonstrate that BMSCs‐free **sGH/PCL** scaffolds could achieve relatively satisfactory bone regeneration, and the regeneration effect could be further improved by loading autologous BMSCs.

## 3 Discussion

In most clinical cases, physiological bone healing commonly undergoes endochondral ossification process because **ECO** mode is accessible to withstand the harsh osteogenic microenvironment of avascular and hypoxic conditions.^[^ ^29^, ^30^, ^31^ ^]^ Inspired by the bone developmental mode of **ECO**, we pioneered a tolerant and rapid endochondral bone regeneration strategy using framework‐enhanced 3D biomineralized matrix hydrogels. First, we prepared 3D biomimetic hydrogels with both hypoxic and osteoinductive microenvironment and followed by integrating 3D‐printed **PCL** framework to improve their mechanical strength and structural fidelity. Importantly, the inherent hypoxic 3D matrix microenvironment effectively activates the HIF‐1α self‐regulation for early‐stage chondrogenesis via TGFβ/Smad signaling pathway due to the obstacle of aerobic respiration. Meanwhile, the strong biomineralized microenvironment could synergistically regulate both bone mineralization and osteoclastic differentiation, and thus accelerate the late‐stage bone maturation. Furthermore, both in vivo ectopic osteogenesis and in situ skull defect repair using BMSCs‐loaded framework‐enhanced biomineralized matrix hydrogels successfully verified the high efficiency and mechanical maintenance of endochondral bone regeneration mode.

To construct ideal scaffolds for realizing bone regeneration via **ECO** mode, it is necessary to provide both hypoxic microenvironment for activating early‐stage chondrogenesis and osteoinductive microenvironment for accelerating late‐stage bone maturation. In the current study, we first revealed that 3D hydrogel culture platform had an inherent hypoxic microenvironment without any extra interference because the highly restricted 3D matrix hydrogels facilitated the BMSCs hypoxic‐inducible self‐regulation and followed by chondrogenic differentiation (upregulation of *HIF‐1α*, *SOX9*, and *COL2*), which is attributable to the innate obstacle of oxygen exchange and vascular infiltration in the dense crosslinked gel network, compared to the oxygen‐rich 2D dish culture model. Meanwhile, the hybrid formulation of **NOIS**, combining hydroxyapatite, calcium silicate, calcium citrate, magnesium phosphate and zinc phosphate, exhibited a strong osteoinductive microenvironment for promoting the upregulation of osteogenic expression of *RUNX2* and *OCN*, which is essential for the acceleration of endochondral bone regeneration. Additionally, mechanical strength and structural fidelity is the next important consideration for realizing bone defect repair via **ECO** mode. The current work continued to use our previously established framework‐enhanced tissue‐engineered bone construction strategy. The results demonstrated that the combination of 3D biomineralized matrix hydrogels with 3D‐printed **PCL** framework could similarly overcome the drawbacks of inferior mechanical properties and morphological fidelity of individual hydrogel scaffolds.

The molecular mechanism of regulating endochondral ossification in 3D biomineralized matrix hydrogels needs to be further determined. As far as we know, it is demonstrated for the first time to uncover the relationship between the regulation of cartilage development and aerobic respiration in hypoxia‐inherent 3D matrix hydrogels. In the current study, the obstacle of oxygen permeation in high‐density 3D matrix hydrogels initially inhibits aerobic respiration (downregulation of *COX1*, *SIRT3*, *MDH2*, and *SDHB*), subsequently leading to the self‐regulation of BMSCs via HIF‐1α signaling pathway.^[^ ^43^, ^44^, ^45^, ^46^, ^47^, ^48^ ^]^ As a result, the biological process of cartilage development was effectively activated, accompanied by the up‐regulated genes of *TGF‐β*, *SMAD3*, *SOX5*/*9*, and *COL2*, which was related to TGFβ/Smad signaling pathway.^[^ ^49^, ^50^, ^51^ ^]^ Predictably, the regenerated cartilage is extremely tolerant to withstand the harsh microenvironment of insufficient angiogenesis and poor nutrient perfusion, which confirmed the reasonability of pre‐chondrogenesis in 3D matrix hydrogels at the early stage. To construct TE bone via **ECO** mode, another consideration is to synergistically provide ideal osteoinductive microenvironment. We exploited the **NOIS**, as a hybrid formulation of multiple biomineralized elements, which could simultaneously upregulate *RUNX2*, *OCN*, *BMP2*/*6*, *OMD*, and *BGLAP* related to bone mineralization,^[^ ^52^, ^53^, ^54^ ^]^ as well as *CALCR*, *OSTM1*, *OSCAR*, and *OCSTAMP* related to osteoclast differentiation.^[^ ^55^, ^56^, ^57^, ^58^ ^]^ The rapid bone maturation is attributed to the efficient achievement of both bone mineralization and remodeling processes involving the deposition of new bone and reabsorption of old bone. Taken together, hypoxic microenvironment provided by high‐density 3D matrix hydrogels and osteoinductive microenvironment supplied by **NOIS** were synergistically regulated BMSC‐based endochondral bone regeneration.

Furthermore, whether framework‐enhanced biomineralized matrix hydrogels could realize both in vivo ectopic osteogenesis and in situ skull defect repair was an important preclinical consideration. The current work demonstrated that BMSCs‐loaded **sGH** groups exhibited rapid and mature endochondral bone regeneration with sufficient bone‐specific ECM deposition, typical bone trabecula structure, and abundant neovascular ingrowth after only four‐weeks implantation. This is mainly dependent on the strong osteogenic effect of **NOIS** integrated with **GH** hydrogels that could effectively promote both bone mineralization and remodeling processes, while BMSC‐loaded **GH** groups still stayed in the cartilaginous stage after four‐weeks implantation, and then gradually evolved into the osteogenic stage after eight‐weeks implantation owing to the subcutaneous vascularized microenvironment in favor of endochondral bone regeneration. Most importantly, the regenerated bone tissue in the BMSCs‐loaded **sGH/PCL** groups showed satisfactory mechanical strength and maintained the structural fidelity due to the mechanical support relied on 3D‐printed framework. Additionally, the critical skull defects of rabbits were successfully repaired using autologous BMSCs‐loaded **sGH/PCL** scaffolds, which was attributed to the advantages of tolerance and high efficiency based on our established endochondral bone regeneration mode, as well as mechanical maintenance by mean of framework‐enhanced strategy. Although the current proposed tissue‐engineered bone strategy has achieved satisfactory repair for rabbit skull defects, the aspects including the effectiveness of larger bone defect repair in a large animal model, complex and precise 3D shape control, as well as the evaluation of biosafety in human body still needs to be further investigated for clinical application in future.

## 4 Conclusion

In conclusion, we proposed a tolerant and rapid endochondral bone regeneration strategy using 3D‐printed **PCL** framework‐enhanced biomineralized matrix hydrogels with both hypoxic microenvironment and osteoinductive microenvironment. The inherent hypoxic microenvironment of 3D matrix hydrogels effectively promotes the positive regulation of cartilage development due to the obstacle of aerobic respiration. Meanwhile, the strong biomineralized microenvironment of **NOIS** could accelerate the mature rate of bone formation. Furthermore, BMSCs‐loaded biomineralized matrix hydrogels successfully achieved ectopic bone regeneration with enough mechanical support and structural fidelity by 3D‐printed **PCL** framework. The current study eventually realized satisfactory skull defect repair by the high‐efficiency endochondral bone regeneration mode, which would offer a promising treatment for craniofacial bone defect repair.

## 5 Experimental Section

### Materials and Animals

In this study, nude mice (six weeks old) were purchased from Shanghai Slaccas Experimental Animal Ltd., and New Zealand white rabbits (2 kg) were purchased from Shanghai Jiagan Biological Technology Co. All experimental protocols involving animals were approved by the Animal Care and Experimental Committee of Shanghai Jiao Tong University, School of Medicine (approval number SH9H‐2021‐A655‐SB).

### Scaffold Preparation

**GelMA**, **HAMA**, and lithium phenyl‐2,4,6‐trimethylbenzoylphosphinate (LAP) were purchased from Huaxiasiyin Biotechnology Co. Ltd. (Shanghai, China). To prepare the **GH** solution, 5% w/v GelMA, 1% w/v HAMA, and 0.2% w/v LAP were dissolved in phosphate‐buffered saline (PBS). This **GH** solution was cross‐linked by UV irradiation (365 nm, 20 mW cm^−2^) for 5 s, resulting in the formation of the **GH** hydrogel. A 5% w/v **NOIS** was prepared by mixing hydroxyapatite (Shanghai Jinsui Biological Technology Co., Ltd., Shanghai, China), calcium silicate (Shanghai Macklin Biochemical Co., Ltd.), zinc phosphate (Shanghai Macklin Biochemical Co., Ltd.), calcium citrate (Shanghai Macklin Biochemical Co., Ltd.), and magnesium phosphate (Shanghai Macklin Biochemical Co., Ltd.) in specific percentages (84%, 10%, 2%, and 2%, respectively). The **NOIS** was added to the **GH** solution to obtain the **sGH** suspension, which was then cross‐linked by UV irradiation to form the **sGH** hydrogel. The addition of **NOIS** to **sGH** and **sPCL** was confirmed using energy‐dispersive X‐ray spectroscopy. **PCL** (80.0 kDa, Aldrich) and **NOIS** were mixed in dichloromethane with a weight ratio of 4:1. This mixture was then printed using a 3D BioArchitect workstation (Regenovo) onto circular meshes with a diameter of 9 mm, 2 × 2 mm^2^ grids, and a thickness of 0.6 mm. Subsequently, the **sPCL** was immersed in the **sGH** suspension to prepare the **sGH/PCL** construct.

### Scaffold Morphological Analysis

The freeze‐dried **GH**, **sGH**, and **sPCL** were sputtered with gold and then examined using an SEM (JEOL JSM‐5600LV, Kyoto, Japan) with an accelerating voltage of 10 kV.^[^ ^59^ ^]^

### Mechanical Tests

The mechanical properties of the **GH**, **sGH**, and **sGH/PCL** scaffolds were assessed using a GT‐TCS‐2000 single‐column apparatus with a 100 N capacity. Briefly, all samples (*n* = 3 per group) were shaped into cylindrical forms (10 mm in diameter and 3 mm in height). A constant compressive strain rate of 1 mm min^−1^ was applied, and the test was terminated at the breaking point of the strain–stress curves. Yang's modulus was then calculated based on the slope of the stress–strain curves for statistical analysis.^[^ ^59^ ^]^

### Rheological Analyses

The rheological analyses were conducted, as previously reported.^[^ ^21^ ^]^ Gel precursors were rheologically tested using a HAAKE MARS III photorheometer with parallel‐plate geometry (P20 TiL, 20 mm diameter) and 365 nm light irradiation (50 mW cm^−2^) at 25 °C.

### Swelling Tests

**GH** and **sGH** hydrogel samples were evaluated after light irradiation (365 nm) using previously reported methods with some modifications.^[^ ^38^ ^]^ Briefly, the hydrogel samples were fully immersed in a PBS solution at 37 °C to perform the swelling test.

### In Vitro Degradation

For the degradation testing, the hydrogel samples (*n* = 3) were placed in a PBS solution econtaining 20 U mL^−1^ of hyaluronidase and 20 U mL^−1^ of collagenase (Worthington Biochemical Corp., USA). The **sPCL** samples (*n* = 3) were placed in a PBS solution with 2000 U mL^−1^ of lipase. Both sets of samples were placed on a shaking table at 37 °C and 120 rpm.^[^ ^21^, ^60^ ^]^

### Rabbit BMSCs Isolation and Culture

Bone marrow from Zealand white rabbits was collected and used to isolate BMSCs. The isolated BMSCs were then cultured in a hypoglycemic medium consisting of Dulbecco's Modified Eagle Medium (Gibco BRL, Grand Island, NY, United States) supplemented with 10% fetal bovine serum (Gibco BRL) and 1% antibiotic/antimycotic (Gibco BRL) and incubated at 37 °C with 5% CO_2_. The expansion of BMSCs was performed using previously reported protocols.^[^ ^61^ ^]^

### Cytotoxicity Tests

To assess the cytotoxicity of **GH** and **sGH**, BMSCs were cultured at a density of 3 × 10^4^ mL^−1^ in the previously described culture medium containing 1% gel precursors. The cell proliferation was assessed after 4 h and 1, 5, and 7 days using the Cell Counting Kit‐8 (Dojindo, Japan). The results were presented as the mean optical density value obtained at a wavelength of 450 nm from five wells, with each experiment repeated three times.^[^ ^62^ ^]^

### Cell Viability

To prepare the **2D‐CP** samples, BMSCs (2 × 10^5^ cells per well), which were cultured using normal culture medium, were seeded in a six‐well plate with glass sheets. BMSCs were then mixed into the hydrogels at a density of 30 × 10^6^ cells mL−^1^ and exposed to 365 nm light irradiation (50 mW cm^−2^) at 25 °C to prepare **3D‐GH** and **3D‐sGH** samples. After in vitro culture for 1 and 7 days, the viability of the BMSCs in the samples was evaluated using the Live/Dead Cell Viability Assay (Invitrogen) and examined using confocal laser scanning microscopy (Leica, TCS SP8 STED 3X).

### Immunofluorescence Staining

For the immunofluorescence assay, the positive expressions of Type II collagen (COL 2, green color) and osteocalcin (OCN, red color) were assessed to further confirm cartilage‐specific and bone‐specific phenotypes, respectively, after 14 days of culture. Meanwhile, 4′,6‐diamidino‐2‐phenylindole staining was used to detect nuclei.

### Real‐Time Quantitative Polymerase Chain Reaction (RT‐qPCR) and Western Blot Analysis

RT‐qPCR was performed to quantify the gene expression levels using β‐actin as a reference gene. Briefly, the samples were harvested into TRIzol (Life Technologies, America), and the total RNA was isolated using the Total RNA Extraction Kit and reverse transcribed to cDNA using the Evo M‐MLV RT Kit for qPCR AG11707 (Accurate Biology, China). Subsequently, the RT‐qPCR reaction was performed using an Applied Biosystems AB instrument (Foster City, CA) to measure the signal intensity. The relative expression levels were quantified using the 2^−ΔΔCt^ method.^[^ ^63^ ^]^ The primers and probes for bone‐related genes (*RUNX2*, *OCN*, *ALP*, *BMP2*, *BMP6*, *OMD*, and *BGLAP*,), cartilage‐related genes (*SOX9*, *COL2*, *TGF‐β*, *SMAD3*, and *SOX5*), aerobic respiration‐related genes (*COX1*, *SIRT3*, *MDH2*, and *SDHB*), osteoclast‐related genes (*CALCR*, *OSTM1*, *OSCAR*, *OSCTAMP*, and *TNFSF11*), *HIF‐1*, and *β‐actin* were designed based on the previously published gene sequences. Total proteins were extracted using a protein extraction kit (Biorab, China). Specific antibodies for bone‐related proteins (RUNX2: 20700‐1‐AP, OCN: DF12303, BMP2: Ab14933, and BMP6: AF5196), cartilage‐related proteins (TGF‐β: BF8012, SMAD3: Ab40854, SOX9: AF6330, COL2: AF0135), and HIF‐1α (BF8002) were used to detect protein expression levels.^[^ ^63^ ^]^

### Eukaryotic Parametric Transcriptome Sequencing Analysis

The samples of **TCP** (**2D**), **GH** (**3D**, **NOIS**−), and **sGH** (**3D**, **NOIS**+) were collected after 14 days of culture and transferred into Invitrogen TRlzol (Thermo Fisher Scientific, Shanghai, China). Total RNA was extracted using TRIzol Reagent (Invitrogen), and genomic DNA was removed using DNase I (TaKara). The RNA samples were then subjected to sequencing using an Illumina HiSeq Xten/Nova (Seq 6000 Sequencing) platform. The raw RNA‐seq reads were aligned to the Oryctolagus cuniculus genome. The mapped reads for each sample were assembled on StringTie using a reference‐based approach.^[^ ^64^ ^]^ The gene expression levels for each transcript were calculated according to the transcript per million reads. Differential expression analysis was performed using DESeq2,^[^ ^65^ ^]^ and a Q‐value of 0.05 or less indicated significant DEGs.

### In Vivo Bone Regeneration

BMSCs‐loaded samples (3 × 10^7^ cells mL^−1^) of **GH**, **sGH**, and **sGH/PCL** were subcutaneously implanted into nude mice. Half of the implanted samples were harvested after four weeks, while the other half were harvested after eight weeks. Additionally, BMSCs‐loaded **sGH/PCL** (3 × 10^7^ cells mL^−1^) and BMSCs‐free **sGH/PCL** samples were implanted into skull defects for 12 weeks, with a blank group serving as a control in this experiment.

### Microcomputed Tomography (Micro‐CT) Analysis

The regenerative samples were harvested and fixed in 4% (w/v) paraformaldehyde for 24 h before being scanned with an X‐ray beam energy of 70 kV and a beam intensity of 114 µA. The final 2D images were loaded into 3D modeling software (Scanco Medical, Switzerland) to quantify the architecture of the samples.

### Histological and Immunohistochemical Analyses

Histological and immunohistochemical analyses were performed to evaluate the regenerated bone in the samples using hematoxylin and eosin, safranin‐O/fast green, and Masson's staining. Immunohistochemical staining of COL2 and OCN was also performed to assess the histological structure and special matrix deposition, respectively. The COL2 and OCN expressions were detected using rabbit polyclonal antibodies against COL2 (ab34712, Abcam, Cambridge, USA) and OCN (GB11022, 1: 500, Servicebio, Wuhan, China), followed by 3,3′‐diaminobenzidine tetrahydrochloride solution. The sections were counterstained with hematoxylin to produce a brown precipitate at the antigenic site. The stained slides were examined and photographed using a microscope (Pannoramic MIDI, 3D HISTECH).

### Quantitative Analysis of the Regenerated Bone

Total DNA, total collagen, and total GAG were quantified to assess cell proliferation and extracellular matrix secretion using a total DNA quantification assay (PicoGreen dsDNA assay, Invitrogen, USA), a total collagen quantification assay (Hydroxyproline Assay Kit, Nanjing Jiancheng Bioengineering Institute, China), and a total GAG quantification assay (dimethylmethylene Blue Assay Kit, Sigma–Aldrich).^[^ ^59^ ^]^

### Projection Area Change Analyses of Regenerated Bone

The round and pentacle projections of the engineered bone were analyzed using ImageJ software (ImageJ 1.53k, National Institute of Mental Health, Bethesda, MD) before and after in vivo implantation. The area of the projections was also calculated using ImageJ software to analyze the characterization of shape maintenaence.^[^ ^66^ ^]^

### Statistical Analysis

Statistical analysis was performed using GraphPad Prism software (Version 5.0), and all data were expressed as means and standard deviations. The student's *t*‐test was used to compare two groups, while one‐way analysis of variance was used to compare more than two groups. A *p*‐value of <0.05 was considered statistically significant.

## Conflict of Interest

The authors declare no conflict of interest.

## Supporting information

Supporting Information

## Data Availability

The data that support the findings of this study are available from the corresponding author upon reasonable request.

## References

[1] Y. Okuchi , J. Reeves , S. S. Ng , D. H. Doro , S. Junyent , K. J. Liu , A. J. El Haj , S. J. Habib , Nat. Mater. 2021, 20, 108. 32958876 10.1038/s41563-020-0786-5

[2] N. Reznikov , M. Bilton , L. Lari , M. M. Stevens , R. Kröger , Science 2018, 360, eaao2189. 29724924 10.1126/science.aao2189 PMC6037297

[3] B. An , Y. Wang , Y. Huang , X. Wang , Y. Liu , D. Xun , G. M. Church , Z. Dai , X. Yi , T.‐C. Tang , C. Zhong , Chem. Rev. 2023, 123, 2349. 36512650 10.1021/acs.chemrev.2c00512

[4] C. Mao , P. Xiao , X.‐N. Tao , J. Qin , Q.‐T. He , C. Zhang , S.‐C. Guo , Y.‐Q. Du , L.‐N. Chen , D.‐D. Shen , Z.‐S. Yang , H.‐Q. Zhang , S.‐M. Huang , Y.‐H. He , J. Cheng , Y.‐N. Zhong , P. Shang , J. Chen , D.‐L. Zhang , Q.‐L. Wang , M.‐X. Liu , G.‐Y. Li , Y. Guo , H. E. Xu , C. Wang , C. Zhang , S. Feng , X. Yu , Y. Zhang , J.‐P. Sun , Science 2023, 380, eadd6220. 36862765 10.1126/science.add6220

[5] Y. Zhang , M. Chen , Z. Dai , H. Cao , J. Li , W. Zhang , Biomater. Sci. 2020, 8, 682. 31776523 10.1039/c9bm01455a

[6] B. Huang , M. Chen , J. Tian , Y. Zhang , Z. Dai , J. Li , W. Zhang , Adv. Healthcare Mater. 2022, 11, 2102540. 10.1002/adhm.202102540 35166460

[7] M. P. Nikolova , M. S. Chavali , Bioact. Mater. 2019, 4, 271. 31709311 10.1016/j.bioactmat.2019.10.005 PMC6829098

[8] G. Battafarano , M. Rossi , V. De Martino , F. Marampon , L. Borro , A. Secinaro , A. Del Fattore , Int. J. Mol. Sci. 2021, 22, 1128. 33498786 10.3390/ijms22031128 PMC7865467

[9] Y. Peng , Y. Zhuang , Y. Liu , H. Le , D. Li , M. Zhang , K. Liu , Y. Zhang , J. Zuo , J. Ding , Exploration 2023, 3, 20210043. 37933242 10.1002/EXP.20210043 PMC10624381

[10] Z. Liu , J. Zhang , C. Fu , J. Ding , Asian J. Pharm. 2023, 18, 100774. 10.1016/j.ajps.2023.100774 PMC9894904 36751654

[11] Y. Zhang , Y. Xu , H. Kong , J. Zhang , H. F. Chan , J. Wang , D. Shao , Y. Tao , M. Li , Exploration 2023, 3, 20210170. 37323624 10.1002/EXP.20210170 PMC10190997

[12] D. Nepal , S. Kang , K. M. Adstedt , K. Kanhaiya , M. R. Bockstaller , L. C. Brinson , M. J. Buehler , P. V. Coveney , K. Dayal , J. A. El‐Awady , L. C. Henderson , D. L. Kaplan , S. Keten , N. A. Kotov , G. C. Schatz , S. Vignolini , F. Vollrath , Y. Wang , B. I. Yakobson , V. V. Tsukruk , H. Heinz , Nat. Mater. 2023, 22, 18. 36446962 10.1038/s41563-022-01384-1

[13] X. Zhang , W. Jiang , C. Xie , X. Wu , Q. Ren , F. Wang , X. Shen , Y. Hong , H. Wu , Y. Liao , Y. Zhang , R. Liang , W. Sun , Y. Gu , T. Zhang , Y. Chen , W. Wei , S. Zhang , W. Zou , H. Ouyang , Nat. Commun. 2022, 13, 5211. 36064711 10.1038/s41467-022-32868-y PMC9445030

[14] M. Li , H. Ma , F. Han , D. Zhai , B. Zhang , Y. Sun , T. Li , L. Chen , C. Wu , Adv. Mater. 2021, 33, 2104829. 10.1002/adma.202104829 34632631

[15] W. Jin , X. Lin , H. Pan , C. Zhao , P. Qiu , R. Zhao , Z. Hu , Y. Zhou , H. Wu , X. Chen , H. Ouyang , Z. Xie , R. Tang , Nat. Commun. 2021, 12, 6327. 34732696 10.1038/s41467-021-26593-1 PMC8566554

[16] X. Wang , V. Agrawal , C. L. Dunton , Y. Liu , R. K. A. Virk , P. A. Patel , L. Carter , E. M. Pujadas , Y. Li , S. Jain , H. Wang , N. Ni , H.‐M. Tsai , N. Rivera‐Bolanos , J. Frederick , E. Roth , R. Bleher , C. Duan , P. Ntziachristos , T. C. He , R. R. Reid , B. Jiang , H. Subramanian , V. Backman , G. A. Ameer , Nat. Biomed. Eng. 2023, 10, 1038. 10.1038/s41551-023-01053-x PMC10804399 37308586

[17] M. Zhou , M. Huang , H. Zhong , C. Xing , Y. An , R. Zhu , Z. Jia , H. Qu , S. Zhu , S. Liu , L. Wang , H. Ma , Z. Qu , G. Ning , S. Feng , Adv. Funct. Mater. 2022, 32, 2200269.

[18] J. Zhang , D. Tong , H. Song , R. Ruan , Y. Sun , Y. Lin , J. Wang , L. Hou , J. Dai , J. Ding , H. Yang , Adv. Mater. 2022, 34, 2202044. 10.1002/adma.202202044 35785450

[19] C. Xu , Y. Kang , S. Guan , X. Dong , D. Jiang , M. Qi , Chin. Chem. Lett. 2023, 34, 107825.

[20] B. Bai , J. Hao , M. Hou , T. Wang , X. Wu , Y. Liu , Y. Wang , C. Dai , Y. Hua , G. Ji , G. Zhou , ACS Appl. Mater. Interfaces 2022, 14, 42388. 36094886 10.1021/acsami.2c08422

[21] J. Hao , B. Bai , Z. Ci , J. Tang , G. Hu , C. Dai , M. Yu , M. Li , W. Zhang , Y. Zhang , W. Ren , Y. Hua , G. Zhou , Bioact. Mater. 2022, 14, 97. 35310359 10.1016/j.bioactmat.2021.12.013 PMC8892219

[22] T. P. Kraehenbuehl , R. Langer , L. S. Ferreira , Nat. Methods 2011, 8, 731. 21878920 10.1038/nmeth.1671

[23] H. M. Kronenberg , Nature 2003, 423, 332. 12748651 10.1038/nature01657

[24] Y. Liu , Z. Yang , L. Wang , L. Sun , B. Y. S. Kim , W. Jiang , Y. Yuan , C. Liu , Adv. Sci. 2021, 8, 2100143. 10.1002/advs.202100143 PMC8188258 34105266

[25] L. Sun , Y. Ma , H. Niu , Y. Liu , Y. Yuan , C. Liu , Adv. Funct. Mater. 2021, 31, 2008515.

[26] P. Cai , S. Lu , J. Yu , L. Xiao , J. Wang , H. Liang , L. Huang , G. Han , M. Bian , S. Zhang , J. Zhang , C. Liu , L. Jiang , Y. Li , Bioact. Mater. 2023, 21, 267. 36157242 10.1016/j.bioactmat.2022.08.009 PMC9477970

[27] H. Niu , Y. Ma , G. Wu , B. Duan , Y. Wang , Y. Yuan , C. Liu , Biomaterials 2019, 216, 119216. 31138454 10.1016/j.biomaterials.2019.05.027

[28] J. He , J. Yan , J. Wang , L. Zhao , Q. Xin , Y. Zeng , Y. Sun , H. Zhang , Z. Bai , Z. Li , Y. Ni , Y. Gong , Y. Li , H. He , Z. Bian , Y. Lan , C. Ma , L. Bian , H. Zhu , B. Liu , R. Yue , Cell Res. 2021, 31, 742. 33473154 10.1038/s41422-021-00467-z PMC8249634

[29] Y. Weng , H. Wang , D. Wu , S. Xu , X. Chen , J. Huang , Y. Feng , L. Li , Z. Wang , Cell Res. 2022, 32, 814. 35821090 10.1038/s41422-022-00687-x PMC9436969

[30] T. Yamada , H. Kawano , Y. Koshizuka , T. Fukuda , K. Yoshimura , S. Kamekura , T. Saito , T. Ikeda , Y. Kawasaki , Y. Azuma , S. Ikegawa , K. Hoshi , U.‐I. Chung , K. Nakamura , S. Kato , H. Kawaguchi , Nat. Med. 2006, 12, 665. 16680148 10.1038/nm1409

[31] T. Saito , A. Fukai , A. Mabuchi , T. Ikeda , F. Yano , S. Ohba , N. Nishida , T. Akune , N. Yoshimura , T. Nakagawa , K. Nakamura , K. Tokunaga , U.‐I. Chung , H. Kawaguchi , Nat. Med. 2010, 16, 678. 20495570 10.1038/nm.2146

[32] H. S. Channey , K. Holkar , V. Kale , G. Ingavle , Biomed. Mater. 2023, 18, 042003. 10.1088/1748-605X/acdb1e 37267985

[33] L. Xiao , M. Wu , F. Yan , Y. Xie , Z. Liu , H. Huang , Z. Yang , S. Yao , L. Cai , Int. J. Biol. Macromol. 2021, 172, 19. 33444651 10.1016/j.ijbiomac.2021.01.036

[34] Q. Li , T. Liu , L. Zhang , Y. Liu , W. Zhang , W. Liu , Y. Cao , G. Zhou , Biomaterials 2011, 32, 4773. 21459437 10.1016/j.biomaterials.2011.03.020

[35] S. Sundelacruz , C. Li , Y. J. Choi , M. Levin , D. L. Kaplan , Biomaterials 2013, 34, 6695. 23764116 10.1016/j.biomaterials.2013.05.040 PMC3724996

[36] J. Diao , J. Ouyang , T. Deng , X. Liu , Y. Feng , N. Zhao , C. Mao , Y. Wang , Adv. Healthcare Mater. 2018, 7, 1800441. 10.1002/adhm.201800441 PMC6355155 30044555

[37] X. Zhao , Y. Hua , T. Wang , Z. Ci , Y. Zhang , X. Wang , Q. Lin , L. Zhu , G. Zhou , Front. Bioeng. Biotechnol. 2022, 10, 916146. 35832408 10.3389/fbioe.2022.916146 PMC9273133

[38] X. Wu , Y. Huo , Z. Ci , Y. Wang , W. Xu , B. Bai , J. Hao , G. Hu , M. Yu , W. Ren , Y. Zhang , Y. Hua , G. Zhou , Appl. Mater. Today 2022, 27, 101478. 10.1016/j.mtbio.2022.100489 PMC9663535 36388453

[39] M. Kanichai , D. Ferguson , P. J. Prendergast , V. A. Campbell , J. Cell. Physiol. 2008, 216, 708. 18366089 10.1002/jcp.21446

[40] L. Chen , X. Huang , H. Chen , D. Bao , X. Su , L. Wei , N. Hu , W. Huang , Z. Xiang , Int. J. Biol. Macromol. 2023, 226, 716. 36526060 10.1016/j.ijbiomac.2022.12.094

[41] J.‐S. Lee , J.‐C. Park , T.‐W. Kim , B.‐J. Jung , Y. Lee , E.‐K. Shim , S. Park , E.‐Y. Choi , K.‐S. Cho , C.‐S. Kim , Bone 2015, 78, 34. 25952967 10.1016/j.bone.2015.04.044

[42] Y. Qiao , X. Liu , X. Zhou , H. Zhang , W. Zhang , W. Xiao , G. Pan , W. Cui , H. A. Santos , Q. Shi , Adv. Healthcare Mater. 2020, 9, 1901239. 10.1002/adhm.201901239 31814318

[43] H. Zeng , J.‐X. Chen , J. Pharm. Pharmacol. 2019, 74, 315. 10.1097/FJC.0000000000000719 PMC6778014 31425381

[44] X.‐H. Wang , S. Xu , X.‐Y. Zhou , R. Zhao , Y. Lin , J. Cao , W.‐D. Zang , H. Tao , W. Xu , M.‐Q. Li , S.‐M. Zhao , L.‐P. Jin , J.‐Y. Zhao , Nat. Commun. 2021, 12, 3428. 34103526 10.1038/s41467-021-23827-0 PMC8187647

[45] S. Moncada , J. D. Erusalimsky , Nat. Rev. Mol. Cell Biol. 2002, 3, 214. 11994742 10.1038/nrm762

[46] W. Liu , E. Qaed , H. G. Zhu , M. X. Dong , Z. Tang , Biomed. Pharmacother. 2021, 141, 111839. 34174505 10.1016/j.biopha.2021.111839

[47] M. R. A. Blomberg , Chem. Soc. Rev. 2020, 49, 7301. 33006348 10.1039/d0cs00877j

[48] P. Altea‐Manzano , A. Vandekeere , J. Edwards‐Hicks , M. Roldan , E. Abraham , X. Lleshi , A. N. Guerrieri , D. Berardi , J. Wills , J. M. Junior , A. Pantazi , J. C. Acosta , R. M. Sanchez‐Martin , S.‐M. Fendt , M. Martin‐Hernandez , A. J. Finch , Mol. Cell 2022, 82, 4537. 36327975 10.1016/j.molcel.2022.10.005

[49] H. Swahn , K. Li , T. Duffy , M. Olmer , D. D. D'lima , T. S. Mondala , P. Natarajan , S. R. Head , M. K. Lotz , Ann. Rheum. Dis. 2023, 82, 403. 36564153 10.1136/ard-2022-223227 PMC10076001

[50] G. Wang , S. Chen , Z. Xie , S. Shen , W. Xu , W. Chen , X. Li , Y. Wu , L. Li , B. Liu , X. Ding , A. Qin , S. Fan , Ann. Rheum. Dis. 2020, 79, 1111. 32409323 10.1136/annrheumdis-2019-216911 PMC7392491

[51] Q. Yao , X. Wu , C. Tao , W. Gong , M. Chen , M. Qu , Y. Zhong , T. He , S. Chen , G. Xiao , Signal Transduction Targeted Ther. 2023, 8, 56. 10.1038/s41392-023-01330-w PMC9898571 36737426

[52] H. Guan , N. Kong , R. Tian , R. Cao , G. Liu , Y. Li , Q. Wei , M. Jiao , Y. Lei , F. Xing , P. Tian , K. Wang , P. Yang , J. Transl. Med. 2022, 20, 132. 35296324 10.1186/s12967-022-03341-7 PMC8925213

[53] W. Lin , X. Zhu , L. Gao , M. Mao , D. Gao , Z. Huang , Cell Death Dis. 2021, 12, 147. 33542209 10.1038/s41419-021-03404-5 PMC7862363

[54] J. T. Rämö , T. Kiiskinen , R. Seist , K. Krebs , M. Kanai , J. Karjalainen , M. Kurki , E. Hämäläinen , P. Häppölä , A. S. Havulinna , H. Hautakangas , R. Mägi , P. Palta , T. Esko , A. Metspalu , M. Pirinen , K. J. Karczewski , S. Ripatti , L. Milani , K. M. Stankovic , A. Mäkitie , M. J. Daly , A. Palotie , Nat. Commun. 2023, 14, 157. 36653343 10.1038/s41467-022-32936-3 PMC9849444

[55] N. Wang , Y. Hao , L. Fu , Nutrients 2022, 14, 3955. 36235607 10.3390/nu14193955 PMC9573743

[56] M. Pata , C. Héraud , J. Vacher , J. Biol. Chem. 2008, 283, 30522. 18790735 10.1074/jbc.M805242200 PMC2662145

[57] N.‐Y. Lin , C.‐W. Chen , R. Kagwiria , R. Liang , C. Beyer , A. Distler , J. Luther , K. Engelke , G. Schett , J. H. Distler , Ann. Rheum. Dis. 2016, 75, 1203. 26113650 10.1136/annrheumdis-2015-207240

[58] W. Feng , Q. Jin , Y. Ming‐Yu , H. Yang , T. Xu , S. You‐Xing , B. Xu‐Ting , C. Wan , W. Yun‐Jiao , W. Huan , Y. Ai‐Ning , L. Yan , T. Hong , H. Pan , M. Mi‐Duo , H. Gang , Z. Mei , K. Xia , T. Kang‐Lai , Biomaterials 2021, 279, 121242. 34768151 10.1016/j.biomaterials.2021.121242

[59] M. Hou , B. Tian , B. Bai , Z. Ci , Y. Liu , Y. Zhang , G. Zhou , Y. Cao , Bioact. Mater. 2022, 13, 149. 35224298 10.1016/j.bioactmat.2021.11.007 PMC8843973

[60] Y. Yang , D. Lei , S. Huang , Q. Yang , B. Song , Y. Guo , A. Shen , Z. Yuan , S. Li , F.‐L. Qing , X. Ye , Z. You , Q. Zhao , Adv. Healthcare Mater. 2019, 8, 1900065. 10.1002/adhm.201900065 30941925

[61] Y. Hua , Y. Huo , B. Bai , J. Hao , G. Hu , Z. Ci , X. Wu , M. Yu , X. Wang , H. Chen , W. Ren , Y. Zhang , X. Wang , G. Zhou , Mater. Today Bio 2022, 17, 100489. 10.1016/j.mtbio.2022.100489 PMC9663535 36388453

[62] M. Hou , B. Bai , B. Tian , Z. Ci , Y. Liu , G. Zhou , Y. Cao , Front. Bioeng. Biotechnol. 2021, 9, 766363. 34993186 10.3389/fbioe.2021.766363 PMC8724709

[63] Y. Huo , Y. Xu , X. Wu , E. Gao , A. Zhan , Y. Chen , Y. Zhang , Y. Hua , W. Swieszkowski , Y. S. Zhang , G. Zhou , Adv. Sci. 2022, 9, 2202181. 10.1002/advs.202202181 PMC9561786 35882628

[64] M. Pertea , G. M. Pertea , C. M. Antonescu , T.‐C. Chang , J. T. Mendell , S. L. Salzberg , Nat. Biotechnol. 2015, 33, 290. 25690850 10.1038/nbt.3122 PMC4643835

[65] M. I. Love , W. Huber , S. Anders , Genome Biol. 2014, 15, 550. 25516281 10.1186/s13059-014-0550-8 PMC4302049

[66] Q. Wang , X. Ran , J. Wang , S. Wang , P. Zhang , E. Gao , B. Bai , J. Zhang , G. Zhou , D. Lei , Adv. Fiber Mater. 2023, 5, 1008.

