# Supplementary material for: Tolerant and Rapid Endochondral Bone Regeneration Using Framework‐Enhanced 3D Biomineralized Matrix Hydrogels
Source: Adv Sci (Weinh). 2023 Dec 21;11(9):2305580. doi: 10.1002/advs.202305580 (PMC10916654; doi:10.1002/advs.202305580)
Supplement: Supplementary file 1 — Supporting Information [file ADVS-11-2305580-s001.pdf]

ADVANCED SCIENCE
Open Access

Supporting Information

for *Adv. Sci.*, DOI 10.1002/advs.202305580

Tolerant and Rapid Endochondral Bone Regeneration Using Framework-Enhanced 3D Biomineralized Matrix Hydrogels

Baoshuai Bai, Yanhan Liu, Jinyi Huang, Sinan Wang, Hongying Chen, Yingying Huo, Hengxing Zhou, Yu Liu, Shiqing Feng, Guangdong Zhou* and Yujie Hua**

Supporting Information

Tolerant and Rapid Endochondral Bone Regeneration Using Framework-Enhanced Three-Dimensional Biomineralized Matrix Hydrogels

Baoshuai Bai, Yanhan Liu, Jinyi Huang, Sinan Wang, Hongying Chen, Yingying Huo, Hengxing Zhou, Yu Liu, Shiqing Feng, Guangdong Zhou,* and Yujie Hua**

B. Bao, **Y. Liu**, **J. Huang**, **S. Wang**, **H. Chen**, **Y. Huo, Y. Liu, G. Zhou**, **Y. Hua**
Department of Plastic and Reconstructive Surgery of Shanghai Ninth People's Hospital
Shanghai Jiao Tong University School of Medicine
Shanghai Key Laboratory of Tissue Engineering, Shanghai 200011, P. R. China.
National Tissue Engineering Center of China, Shanghai 200241, P. R. China.
E-mail: G. Zhou: guangdongzhou@126.com; Y. Hua: yujiehua@shsmu.edu.cn
B. Bao, **H. Zhou, S. Feng**
Department of Orthopaedics, Qilu Hospital of Shangdong University Centre for Orthopaedics, Advanced Medical Research Institute, Shandong University, Jinan, Shandong 250100, P. R. China.
Department of Orthopaedics, The Second Hospital of Shandong University, Cheeloo College of Medicine, Shandong University, Jinan, Shandong 250033, P.R. China.
E-mail: S. Feng: shiqingfeng@sdu.edu.cn
Y. Liu
Department of Ophthalmology, Renji Hospital, School of Medicine, Shanghai Jiao Tong University, Shanghai 200127, China.

Keywords: bone regeneration; endochondral ossification; hydrogels; hypoxic microenvironment; biomineralization

Figures

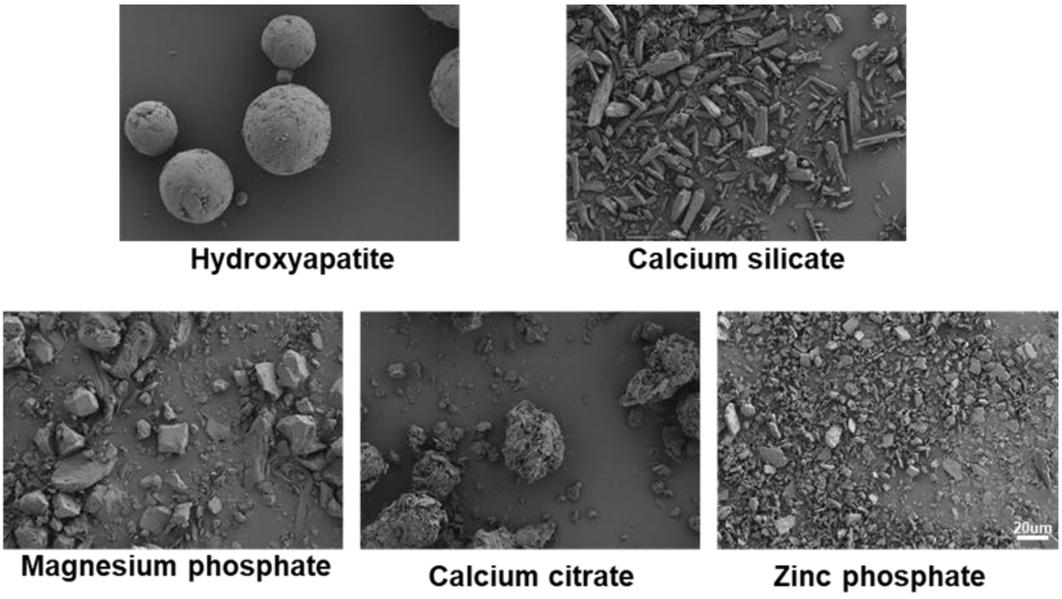

Figure S1. The SEM images of hydroxyapatite, calcium silicate, magnesium phosphate, calcium citrate, and zinc phosphate.

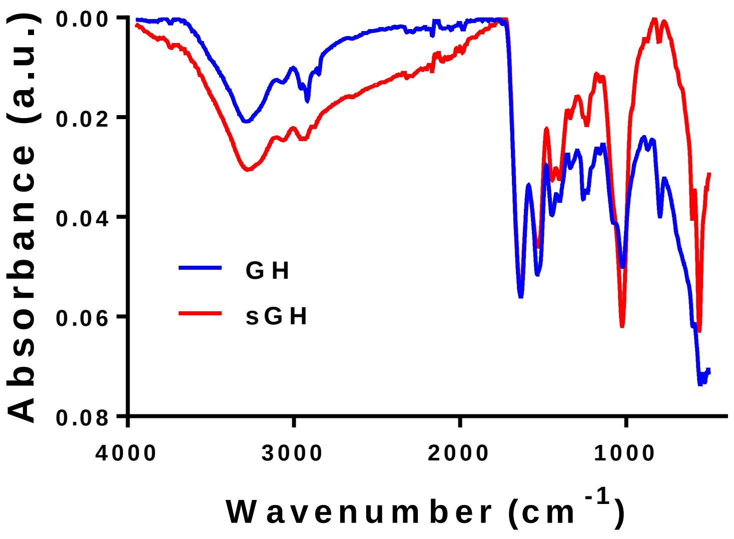

Figure S2. The Fourier transform infrared spectroscopy (FTIR) of GH and sGH hydrogels.

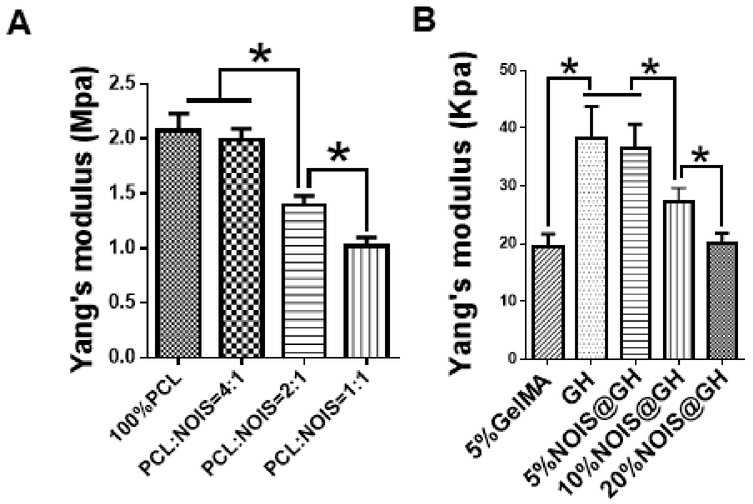

Figure S3. Yang's modulus of sGH and sPCL with different contents of NOIS.

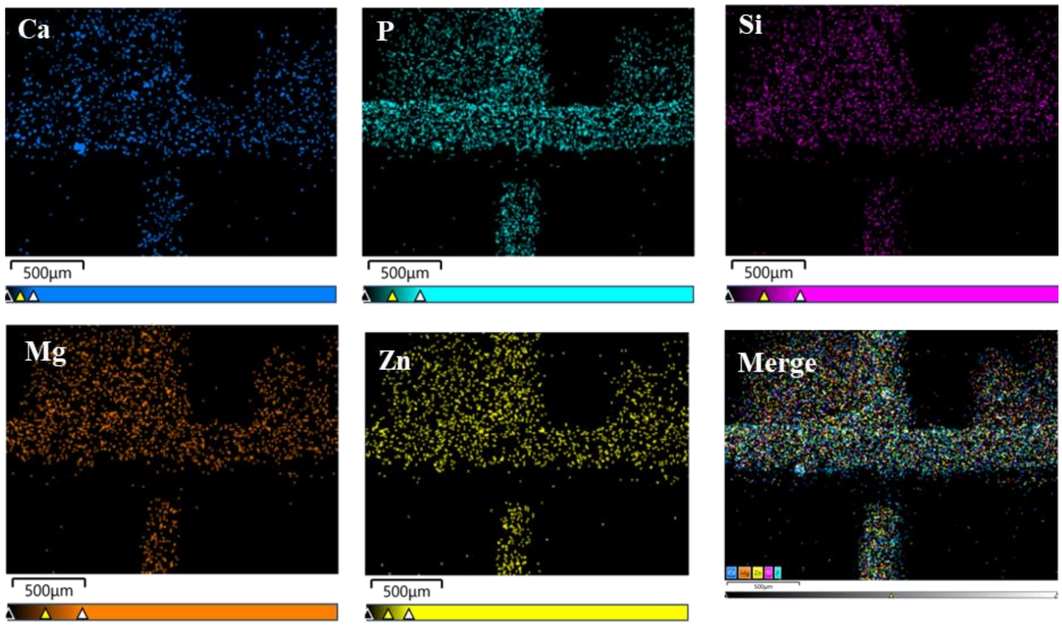

Figure S4. Elemental analysis mapping of 3D printed sPCL.

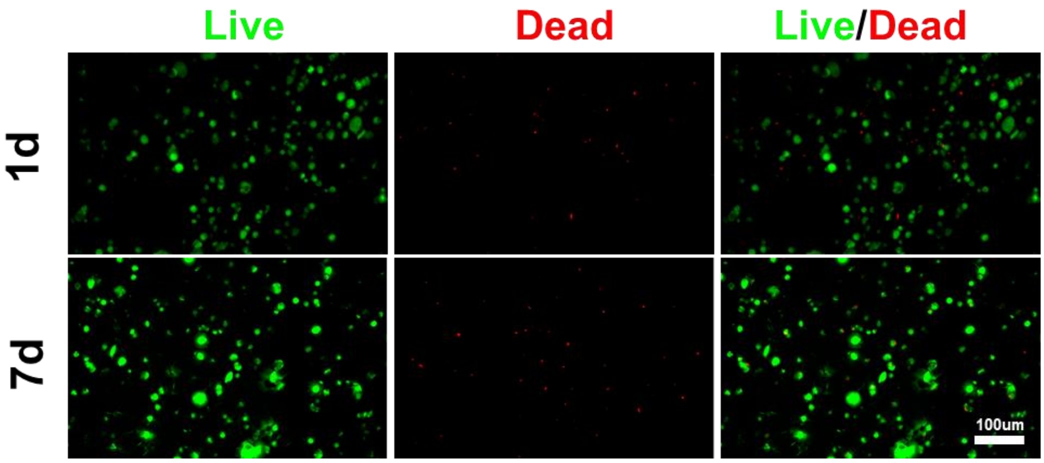

Figure S5. Live/dead staining of BMSCs in the GH hydrogels after 1- and 7-days culture *in vitro*.

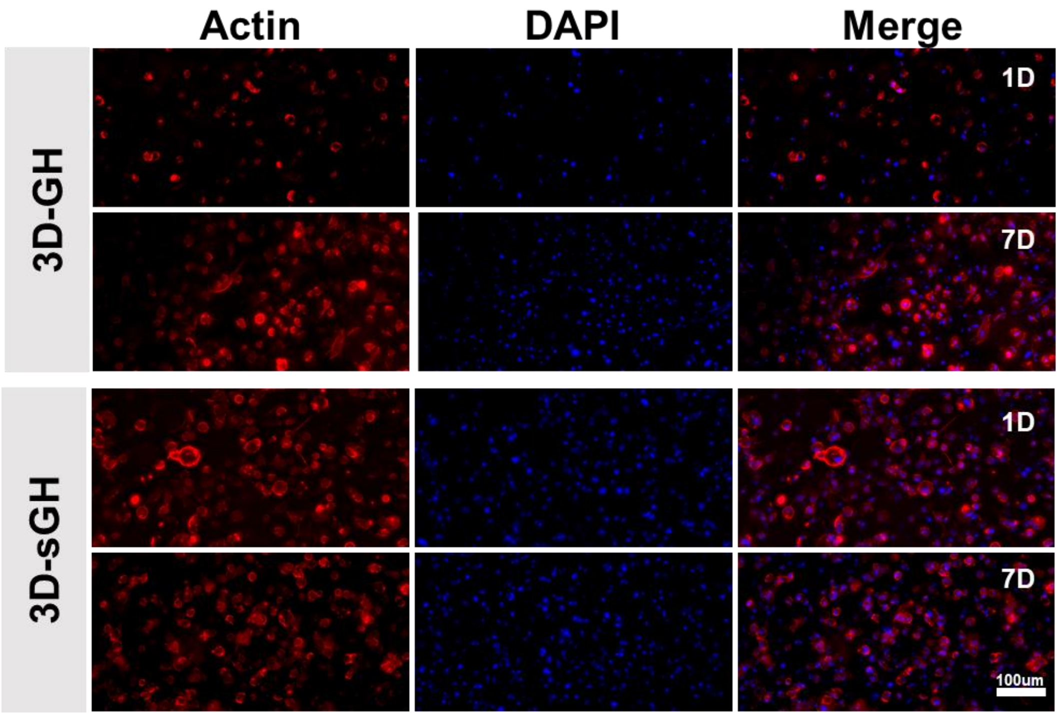

Figure S6. The cytoskeleton staining of BMSCs in 3D-GH and 3D-sGH hydrogels after 1- and 7-days culture *in vitro*.

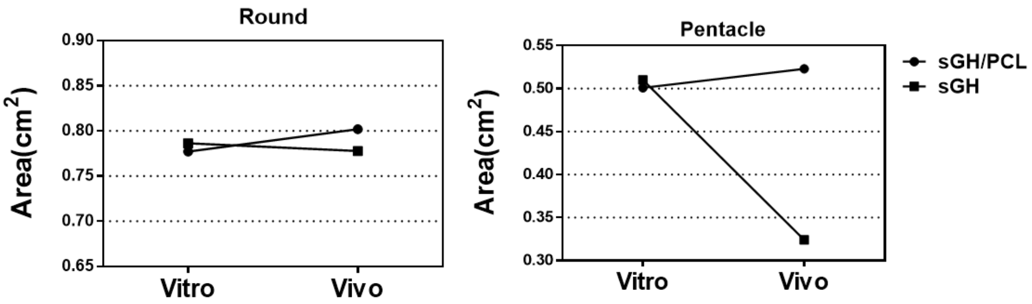

Figure S7. The Change in area after *in vivo* implantation of sGH/PCL and sGH groups. Round hydrogels changed lightly without enhanced framework, while the pentacle hydrogel changed significantly.

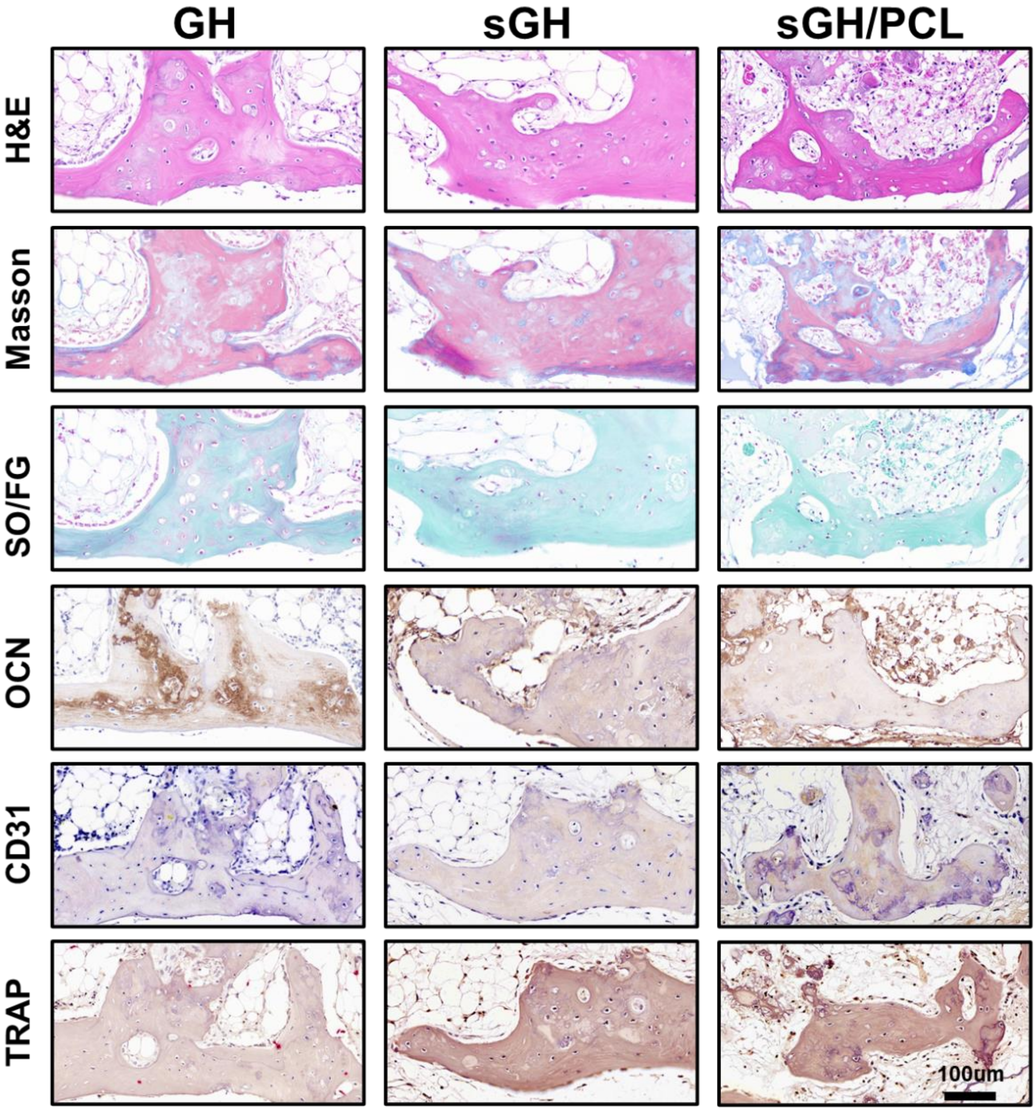

Figure S8. Evaluation of regenerated bone in nude mice at 8 weeks. The results of staining showed similarly regenerated bone of **GH**, **sGH**, and **sGH/PCL** groups.

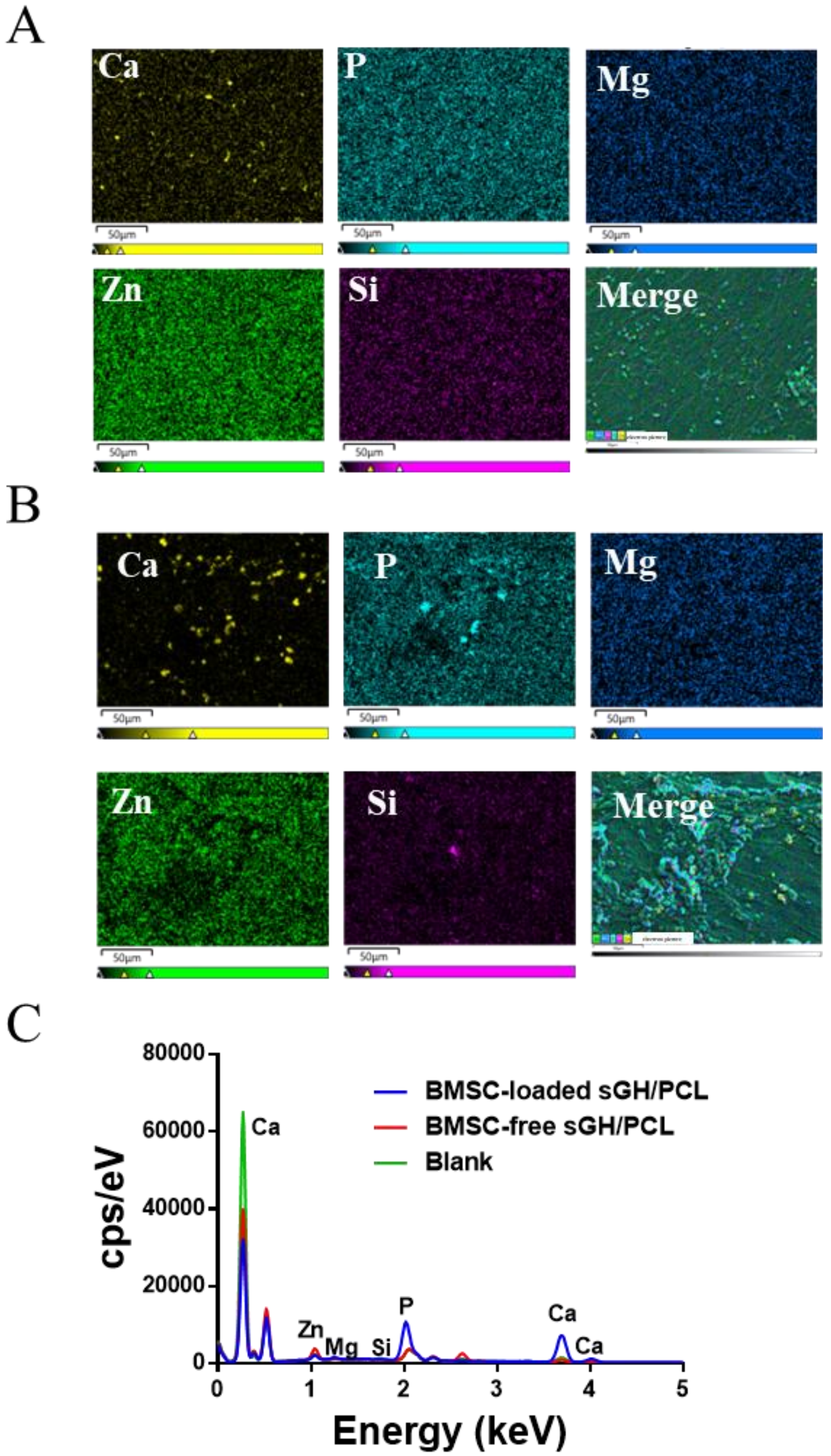

Figure S9. **The elemental analysis (Ca, P, Mg, Zn, Si distribution) of BMSCs-free sGH/PCL (A, C), and blank groups (B, C).**
